# Verification of correction factors for determining mean annual levels of radon in underground facilities

**DOI:** 10.1007/s10653-024-02132-x

**Published:** 2024-07-29

**Authors:** Lidia Fijałkowska-Lichwa, Jerzy Olszewski

**Affiliations:** 1 Faculty of Civil Engineering, Wrocław University of Science and Technology, Wybrzeże S. Wyspiańskiego 27, 50−370 Wrocław, Poland; 2 Department of Radiation Protection, Nofer Institute of Occupational Medicine, Św. Teresy 8, 91−348 Łódź, Poland

**Keywords:** Monthly and quarterly correction factors, Radiology database, Mean annual radon concentration, Underground facilities, Workplaces

## Abstract

The first verification of a tool developed to improve the work of controlling bodies, managers and employees of underground facilities subject to radiation protection requirements was conducted. The recommended values of correction factors were verified using archival results of measurements conducted for the Institute of Occupational Medicine in Łódź in seven underground workplaces in Poland over exposure periods of a month (10,8678 data) and a quarter of a year (53,688 data). In a cave two groups of monthly factors, produced estimates with almost 70% to 99% consistency with the measured values. Along tourist routes located in mines, a similar fit was obtained using three groups of correction factors for measurement results from March, June and July. In the extraction areas of active underground mines, the best fit was produced by factors calculated as averages for spaces varying in the degree of insulation and ventilation method, while in other departments of mining plants, by correction factors recommended for facilities equipped with mechanical ventilation systems. All the quarterly correction factors produced the best fit between estimated mean annual concentrations and measurement results obtained in the second quarter of the calendar year. A wide variation in result consistency (from 20–30 to 65–80%) obtained for two underground tourist routes in the fourth quarter of the year demonstrates that it is best not to adopt results from this measurement period (October-December) for estimating mean annual radon concentration using the set of quarterly correction factors.

## Introduction

In Poland, provisions concerning radon in workplaces are regulated by an amendment to radiological protection laws (Law 2000; Directive 2013). In 2019, the reference level of radioactive radon in the air of workplaces was defined as 300 Bq/m^3^ (Article 23b of the Law Journal of Laws 2023 item 1173 consolidated text). The responsibility for conducting measurements necessary for determining the mean annual value of radon concentration rests with the head of the entity carrying out business activity (Art. 23c of the Law 2000). In the case of workplaces located in areas where mean annual radioactive concentration of radon may exceed the reference level in a significant number of buildings, legislation specifies the location of these workplaces (on the ground floor or at basement level) and the character of their activity (related to groundwater treatment). A list of workplaces exposed to increased radon concentrations, in addition to those specified by Article 23 of the Atomic Law, has been published in the National Action Plan for Long-Term Risks of Radon Exposure in Buildings and Workplaces (Minister of Health Announcement 2021). Apart for defining a duty to conduct measurements of radon concentration, the law obliges entity heads (employers) to optimise the exposure of employees working in such conditions, and in the event of an exceedance of the level of 300 Bq/m^3^ a year, to take measures ensuring the reduction of workers’ exposure to radon. In Poland, radon measurements in workplaces can be performed by accredited dosimetry laboratories (5 centres in total) and conditionally (until the end of 2023), laboratories which have obtained satisfactory results in intercomparison measurements organized by the Chief Sanitary Inspector (GIS). Soon, such limited technical backup may become an obstacle to providing reliable measurement results, which are subject to strict control by regional GIS units.

A partial solution, but available for quick implementation, is assessment of mean annual levels of radon concentration in workplaces by using correction factors calculated for one-month and three-month (quarter) measurement periods. They have been developed in compliance with the Chief Sanitary Inspector (GIS) guidelines and the provisions of the National Action Plan (Minister of Health Announcement of 22 January 2021 Item 169) by employees of Wrocław University of Science and Technology (Fijałkowska-Lichwa & Przylibski, 2022). The solution, enabling estimation of mean annual values of radon concentration in various types of underground facilities, was developed on the basis of more than 440,000 radon concentrations registered at 1-h intervals in several well-known underground workplaces in the Sudetes. Based on their analyses, the researchers created a database of monthly (k_1m_) and quarterly (k_3m_) correction factors recommended for use in many countries in Europe and worldwide located in zones of temperate climate with continental-to-oceanic (marine) transition, with four distinct seasons, i.e. spring, summer, autumn and winter.

In their study, Fijałkowska-Lichwa and Przylibski (2022) demonstrated relationships between the ranges of variation in estimated correction factors k_1m_ and k_3m_ and individual characteristics of underground facilities such as air exchange method and the extent to which their interiors are insulated from the atmosphere. As a result, they grouped the correction factors by matching them to facilities without a specified ventilation method and those with mechanical and natural ventilation, including spaces well insulated from the atmosphere. Fijałkowska-Lichwa and Przylibski (2022) emphasized that the estimation accuracy of mean annual levels of radon concentration in underground facilities depends on the adopted type of correction factor (k_1m_ or k_3m_). They recommended using monthly correction factors (k_1m_) for determining mean annual concentration in facilities with natural air exchange and quarterly factors (k_3m_) for mechanically ventilated spaces. They specified the value range of k_1m_ factors (from 1.2 to 3.3) for the first group between January and March and between October and December. In the other months of the calendar year, k_1m_ stays in the range of 0.6 to 0.8. In facilities with undisturbed natural indoor-outdoor air exchange, k_1m_ varies from 1.0 to 1.5 between January and March and between September and December, and from 0.7 to 0.8 between May and September. In April, it takes the value of 1.0. For facilities with mechanical ventilation, k_3m_ is characterized by values in the range 1.3–1.4 in the first and the fourth quarter of the year, and of 0.9 in the second and the third quarter. In facilities with unknown ventilation methods or when mixed-mode ventilation is used, Fijałkowska-Lichwa and Przylibski (2022) suggested using mean values. For k_1m_ they range ≥ 1.0–1.6 (from January to March and from September to December) and < 1.0–0.7 from April to November. For k_3m_ they range 1.3–1.4 (in the first and the fourth quarter), and equal 0.9 (in the second and the third quarter). The results of Fijałkowska-Lichwa’s and Przylibski’s analyses (2022) show that the best consistency of estimated and measured mean annual values are obtained based on correction factors estimated in March (over 60%), August (over 70%) and September (even over 80%), and the worst—based on data collected between October and December. Such results have confirmed that the accuracy of correction factors, both for monthly and quarterly exposures, is much higher in periods of increased radon activity concentration. Such conclusions have explicitly underscored the relevance of the proposed solution for broadly defined radiological protection.

In view of the need to determine the mean annual level of radon concentration for initial assessment of occupational exposure risk, the authors went on to verify the solution proposed by Fijałkowska-Lichwa and Przylibski (2022). The rationale behind and the need for such analyses lie in the fact that so far it has been the only alternative solution described in Polish and world literature to streamline the time-consuming measurement process, facilitate corrective decisions and recommend strategic remedial actions aimed at keeping radon concentrations in underground workplaces at a safe level of 300 Bq/m^3^. The authors believe that the alternative solution proposed by Fijałkowska-Lichwa and Przylibski (2022) can be regarded as a good practice relating to methods and techniques of recognizing the first signs of risk of occupational exposure to radon and its short-lived progeny.

So far, databases of correction factors available in literature have been compiled only for dwellings in Poland (Kozak et al., 2011), Ireland (Burke & Murphy, 2011), and Great Britain (Gillmore et al., 2005; Groves-Kirby et al., 2015), in South Korea (Park et al., 2018), several provinces in Canada (Krewski et al., 2005), Pakistan (Rahman et al., 2007) and Slovakia (Müllerová et al., 2022). It is still just a fraction of all places in the world at risk of increased exposure to radon and its short-lived daughters.

## Material and methods

To carry out their research task, the authors used a database of archival radiology data from an accredited dosimetry laboratory at the Institute of Occupational Medicine in Łódź with accreditation No. AB 327 of the Polish Center for Accreditation for radon (Rn-222) activity concentration measurements made in any range of time exposure by track detectors (20–4300 kBq∙h∙m^−3^).

These are the results of radon concentration measurements conducted in workplaces between 1995 and 2012, partly published in Polish research papers (Olszewski, 2006; Olszewski et. al. 2010, 2015) and international publications (Olszewski et al., 2005; Walczak et al., 2017). The workplaces chosen for this study satisfy Fijałkowska-Lichwa’s and Przylibski’s (2022) criteria concerning the ventilation method and the degree of insulation from the atmosphere. A new aspect is the work time schedule. In the case of tourist routes, work was done from 9 a.m. to 6 p.m. 7 days a week (it was shortened in autumn and winter). As for the mining plants, work was performed in a four-on four-off shift pattern seven days a week, except a total of 2–5 days a year at Christmas, Easter and on public holidays.

Measurements in underground tourist facilities were conducted in accordance with recommendations issued by the International Commission on Radiation Protection (ICRP 2011, 2014, 2017), the International Atomic Energy Agency (IAEA 2014), the International Radon Measurement Association (IRMA 2017), and the Chief Sanitary Inspectorate (GIS) in Poland (Report 2021). The measurements were conducted using track detectors with a diffusion chamber. The number of used CR-39 track detectors varied as it was adjusted individually to the surface areas of the spaces housing particular workplaces. The detectors were spaced evenly in places occupied by employees, surveillance staff or service workers for at least 4 h a day. Measurements were also carried out in mine chambers, machine rooms, welfare facilities such as bathrooms or changing rooms, reception areas, corridors and entrance/exit halls in tourist facilities located on the ground floor or at basement level. In such places, there was a risk of sizeable radon ingress, and employees occupied them for at least 50 h a year, i.e. about 1 h a week. Along tourist routes, detectors were placed at a height of the breathing zone (1.5 to 2 m above the floor) at places of longer stops representative of the whole space.

In active mining plants, radon concentration was measured using a method consistent with an original methodology for assessing radiation exposure developed entirely by employees of the Radioactive Contamination Department of the Institute of Occupational Medicine in Łódź. The basic tool for measuring radon concentration in underground mines was the track detector LR-115 by French manufacturer Kodak Pathe placed in an open cassette type OC-1, which was mounted directly on mining helmets. It is the so-called HCS system (Helmet Cassette System) based on employees wearing helmets with the cassettes (sealed after the insertion of a detector to prevent unauthorised opening) for a determined exposure time. A detailed description of the measurement system and the dosimetric technique of reading track density from the detector has been provided by Domański et al., (1981).

The method of estimating mean annual values of radon concentration used by the authors was identical to the assumptions presented by Fijałkowska-Lichwa and Przylibski (2022). Two relations were used to determine the mean annual concentration. For measurements in one-month exposure, the mean annual value of radon concentration was estimated using Eq. (1), and for 3-month (quarterly) exposure—according to relation 2:

1 $$C_{Rn} = k_{1m} \cdot C_{1mRn}$$

where C_Rn_ is the mean annual activity concentration expressed in Bq/m^3^, k_1m_ is the correction factor for the month of the exposure, C_1mRn_ is the mean monthly value of ^222^Rn activity concentration expressed in Bq/m^3^, and

2 $$C_{Rn} = k_{3m} \cdot C_{3mRn}$$

where k_3m_ is the correction factor for three month exposure, and C_3mRn_ is the mean value of ^222^Rn activity concentration for a quarter of a year, expressed in Bq/m^3^.

**Fig. 1:**
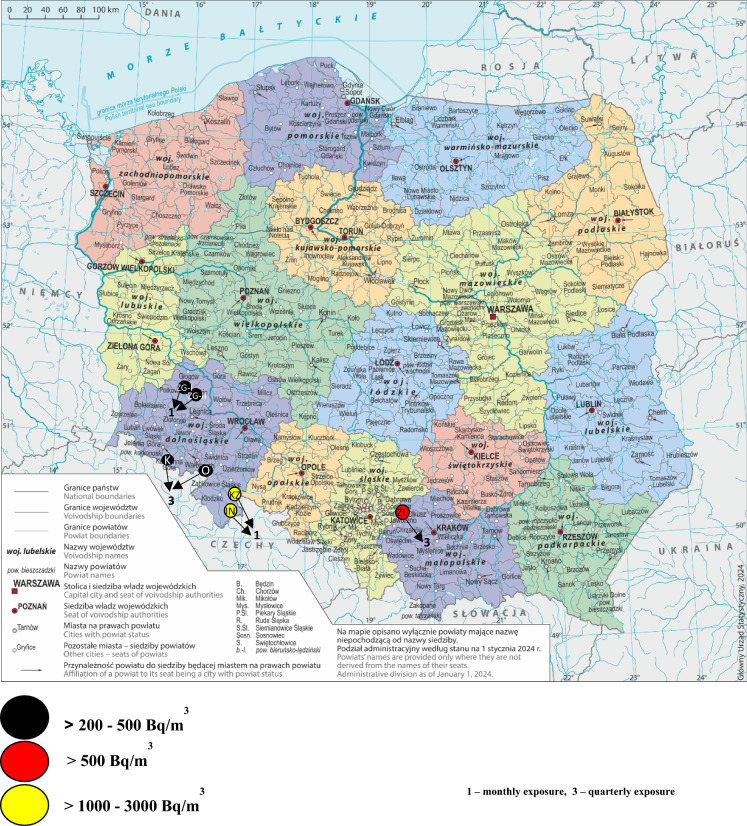
Location of selected underground facilities on a digital administrative map of Poland prepared by the Head Office of Geodesy and Cartography (GUGiK) on the basis of the General Geographic Objects Database available on Statistics Poland website (https://stat.gov.pl/statystyka-regionalna/jednostki-terytorialne/podzial-administracyjny-polski/?pdf=1) with indicated mean annual values of radon concentration determined on the basis of radiation measurements conducted from 1995 to 2012 for the Institute of Occupational Medicine in Łódź (Authors’ own work). Explanation: ZG—copper mining plants, I, II—mining regions, ZL—zinc-and-lead mining plant, K—inhalatorium and tourist rote in Kowary, O- military facility Osówka, JN—Niedźwiedzia (Bear) Cave in Kletno, KZ- tourist route in Gold Mine in Złoty Stok. The map available on the office's website presents the administrative division of Poland into voivodeships and counties on January 1, 2024. Its purpose is to make information available to a wide range of recipients, including: public administration, entrepreneurs, individual users and scientific and research institutions. The authors used it as an illustrative map for scientific and research data

The mean annual value was estimated for four groups of monthly correction factors in four underground facilities and for three groups of quarterly correction factors in three underground facilities (Table 1, Fig. 1).

**Table 1:** Overview of correction factors: 4 monthly groups (a) and 3 quarterly groups (b) Authors’ own work based on Fijałkowska-Lichwa and Przylibski (4)

Time of exposure	k_1ma_—for naturally ventilated facilities well insulated from the atmosphere	k_1mb_—for non-insulated facilities	k_1mc_—averaged for underground facilities	k_1md_—for facilities with mechanical ventilation system
Month (a)				
January	3.34	1.41	1.52	0.73
February	2.80	1.38	1.42	0.38
March	1.56	1.19	1.16	0.52
April	0.55	0.96	0.88	0.40
May	0.83	0.77	0.74	0.40
June	0.60	0.71	0.70	0.73
July	0.70	0.71	0.81	1.76
August	0.56	0.69	0.78	1.80
September	0.63	0.83	0.95	2.40
October	1.40	1.00	1.10	1.20
November	1.20	1.30	1.30	1.60
December	2.70	1.50	1.60	1.60
Quarter (b)	k_3ma_—for naturally ventilated facilities	k_3mb_—for facilities with a mechanical ventilation system	k_3mc_—averaged for all underground facilities	
No. 1	0.62	1.40	1.32	
No. 2	0.76	0.81	0.90	
No. 3	2.14	0.72	0.87	
No. 4	1.96	1.31	1.37

## Results and discussion

The estimation accuracy of mean annual radon concentrations calculated using four groups of monthly correction factors was verified with data obtained from measurements conducted for many years in the cave, a former mine operating as an underground tourist route, in two active copper mines and one disused (since 2020) zinc and lead mine (Fig. 1). The correction factor k_1md_ was excluded from analyses conducted for the cave because of its lack of mechanical ventilation. The fitting accuracy of quarterly factors was checked against the results of radon measurements conducted in the workings of a former mine used as a tourist route and a radon inhalatorium, and in a tourist-accessible former military facility in the Sudetes. A list of the groups of correction factors used in the comparative analyses is provided in Table 1.

### One-month data

**Fig. 2:**
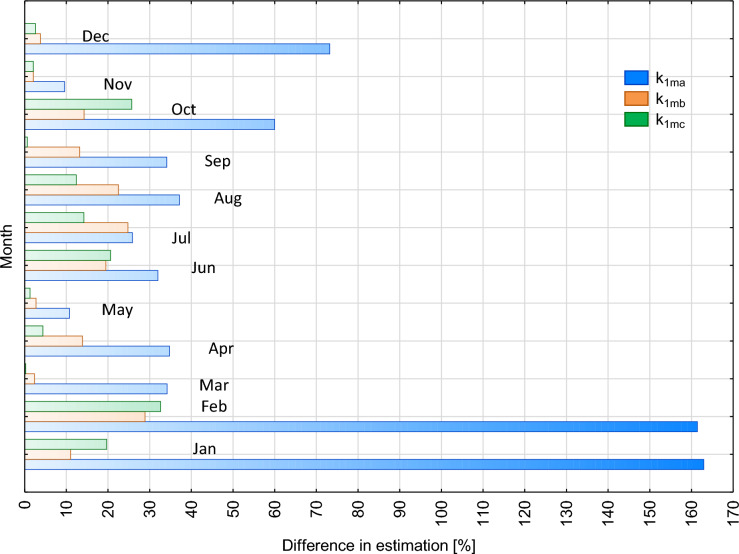
Differences in fits of mean annual measured value of radon concentration in a tourist facility (cave) to values estimated using correction factors k_1m_. Explanation: k_1ma_—monthly correction factor for naturally ventilated facilities well insulated from the atmosphere, k_1mb_—monthly correction factor for non-insulated facilities, k_1mc_—averaged monthly correction factor for underground facilities, colours of legend and bars are the same, for bars used a colour blur

Verification of Fijałkowska-Lichwa’s and Przylibski’s (2022) assumptions started with organising measurement data in one-month intervals. The mean values obtained for successive months were converted according to Eq. 1 using monthly correction factors (Tab. 1a) for: naturally ventilated spaces well insulated from the atmosphere (k_1ma_), naturally ventilated spaces without insulation (k_1mb_) and all facilities in total (k_1mc_). Data analysis was performed using measurement results from 865 points. On the basis of nearly 10 years of measurements, the mean annual value of radon concentration in the cave was calculated as 2034 Bq/m^3^. The values of radon concentration in the cave estimated by using monthly correction factors varied (Fig. 2). The best fit to the annual average was obtained in the cave using correction factors k_1mb_ and k_1mc_ throughout all calendar year. At the same time, correction factors k_1ma_ produced comparable results only in November and May. The mean annual values of radon concentration estimated on the basis of measurements in the indicated months differed by less than 10% compared to the value determined using the other two groups of factors and the value resulting from measurements. The lowest accuracy compared to the mean measured annual concentration was obtained in the cave using the k_1ma_ factor in October and between December and February. In this period, the estimated mean annual concentration differed by 40% to even more than 160% from the value obtained from measurements (Fig. 2).

**Fig. 3:**
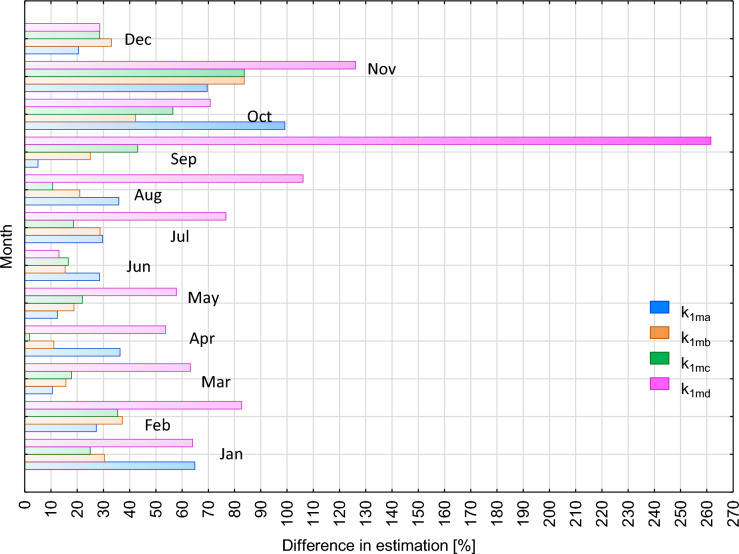
Differences in fits of mean annual measured value of radon concentration in a tourist facility located in a mine to values estimated using correction factors k_1m_. Explanation: k_1ma_—monthly correction factor for naturally ventilated facilities well insulated from the atmosphere, k_1mb_—monthly correction factor for non-insulated facilities, k_1mc_—averaged monthly correction factor for underground facilities, k_1md_—monthly correction factor for facilities with mechanical ventilation system, colours of legend and bars are the same, for bars used a colour blur

The same kind of analysis was conducted for the correction factors k_1m_ used to calculate the mean annual radon concentration in an underground tourist route operating in a former mine in Złoty Stok. The analyses comprised measurement results from 90 measurement points. On the basis of measurements conducted between September 2004 and January 2006, the mean annual concentration of radon in the whole facility was determined to be 1434 Bq/m^3^. The mean annual values of radon concentration estimated by using monthly correction factors varied (Fig. 3). The best fit of the estimated value to the mean annual value derived from measurements was observed in March, May, June and July for three k_1m_ correction factors: k_1ma_, k_1mb_, and k_1mc_. In these months, the estimated values differed by < 10% to 30% from the mean annual value derived from measurements. The worst fit of the estimated value was obtained in October and November for all the four groups of k_1m_ correction factors (Fig. 3). The k_1md_ correction factors were characterized by a much lower fit. In February, March, April, May, August, and November, the estimated annual means differed by two and even four times from the values calculated using the other three groups of factors (Fig. 3). A comparable degree of fit for all correction factors was observed in December and June (Fig. 3).

Analyses verifying the k_1m_ correction factors recommended for determining the mean annual value of radon concentration were also conducted in the workings of metal ore mines. In total, these were 4 divisions of a copper mining company and 9 divisions of a zinc and lead mine. In both cases, mean annual values were determined using k_1md_ correction factors for underground facilities equipped with a mechanical ventilation system, and k_1mc_ factors, calculated as averages, for all underground facilities. The estimations were based on 1-month measurements conducted in the copper mining company in February 2012, and in the zinc and lead mining company—in December 2006. Based on environmental dosimetry measurements conducted on a group of 4254 employees of copper mine No. 1 and 3037 employees of mine No. 2 (including 1228 employees in the main area, 1092 in the eastern area, and 717 in the western area of mine No. 2), and 1312 employees of the zinc and lead mining company (including 402 in mine No. 1, 45 in mine No. 3, 444 in mine No. 6, 53 in mine No. 8, 64 in mine No. 9, 113 in the mining and shaft works department, 40 in the electrical department, 38 in the mechanical department, 77 in the shaft and heavy machinery depot and 36 in the ventilation department), mean annual concentrations of radon in each of these plants were determined. For the copper mines these were: 484 Bq/m^3^ (mine No.1), and 420 Bq/m^3^ (mine No. 2). For the zinc and lead mining plant it was 857 Bq/m^3^.

**Fig. 4:**
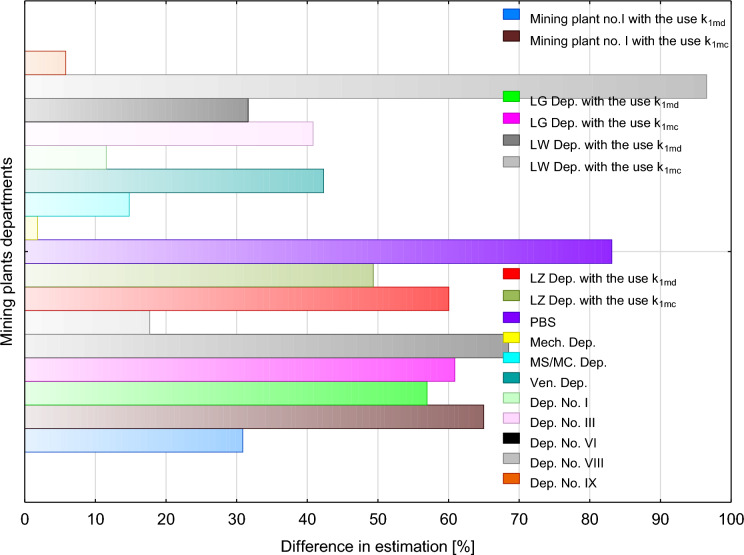
Differences in fits of mean annual measured value of radon concentration in metal ore mines in Poland to values estimated using monthly correction factors k_1m_ recommended by Fijałkowska-Lichwa and Przylibski (4). Explanation: k_1mc_—averaged monthly correction factor for underground facilities, k_1md_—monthly correction factor for mechanically ventilated facilities. Explanation: ZG—mining plant, LG- main area of copper mine No. 2, LZ—western area of copper mine No. 2, LW—eastern area of copper mine No. 2, I, III, VI, VIII, IX—parts of zinc-and-lead mining company, MS/MC—shaft and heavy machinery depot, Mech—mechanical department, PBS—mining and shaft works department, Vent.—ventilation department, k_1mc_ = 0.38 in February and 1.60 in December, k_1md_—1.42 in February, 1.60 in December. In copper mining plants, measurements were conducted in February 2012, and in zinc-and-lead mining plant—in December 2006, colours of legend and bars are the same, for bars used a colour blur

After applying the k_1m_ correction factors, it turned out that the best fits were obtained in the mechanical department, mine No. 9 and mine No. 1 of the zinc and lead mining plant (even 90% consistency of results). In copper mine No. 1, the consistency of results reached 70%. At the same time, in all the extraction areas of copper mine 2, it ranged from just above 40% in the main and the western area to just below 70% in the eastern area (Fig. 4). Outside the western area, better fits were obtained when using k_lmd_ correction factors. In the western area, the difference between the estimate obtained by using the k_1md_ factor and that obtained with k_1mc_ was just above 10%. A higher accuracy was obtained for the correction factor k_1mc_ (Fig. 4).

### Three-month data

In Kowary, verification of the correction factors was performed using three-month data. Along the tourist route, measurements were conducted at a total of 72 points from November 2000 to 31 January 2005. In the inhalatorium, measurements were conducted at 52 measurement points between August 2001 and 31 January 2005. The mean annual radon concentrations derived from the results of these measurements were 391 Bq/m^3^ for the tourist route and 690 Bq/m^3^ in the inhalatorium. For successive quarters of the calendar year, mean annual concentrations of radon were estimated using three groups of k_3m_ correction factors recommended for: naturally ventilated facilities (k_3ma_), facilities with mechanical ventilation systems (k_3mb_) and all facilities regardless of the ventilation method and the degree of insulation (k_3mc_).

**Fig. 5:**
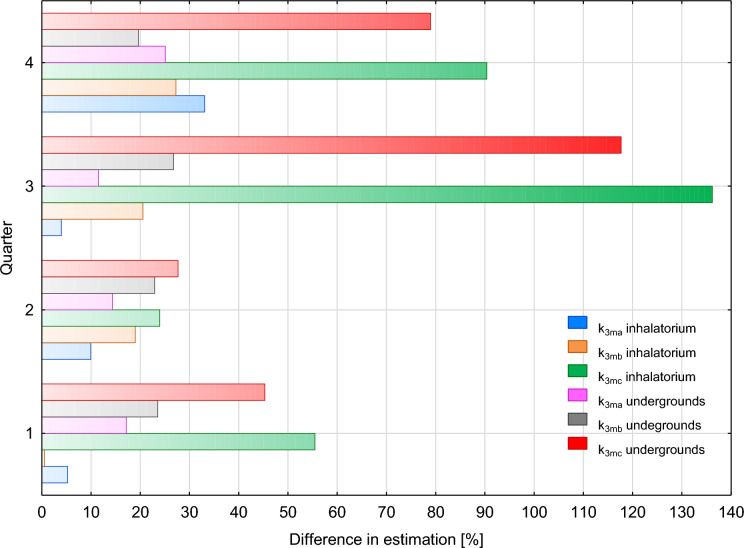
Differences in fits of mean annual measured values of radon concentration in workings of a former uranium mine in Kowary operating as a tourist route and radon inhalatorium to values estimated using quarterly correction factors k_3m_ recommended by Fijałkowska-Lichwa and Przylibski (4). Explanation: k_3ma_—quarterly correction factor for naturally ventilated facilities, k_3mb_ quarterly correction factor for facilities with a mechanical ventilation system, k_3mc_—averaged quarterly correction factor for all underground facilities. Three-month periods corresponded to four quarters of calendar year. Exposure: 1st quarter: from 2160 to 2856 h, 2nd quarter: from 1872 to 2208 h, 3rd quarter: from 2208 to 2616 h, 4th quarter: from 2208 to 2592 h, colours of legend and bars are the same, for bars used a colour blur

In the whole facility, the closest to the mean annual value derived from measurements was obtained in the second quarter of the calendar year (from > 70 to 82%) using all the groups of k_3m_ correction factors. The worst fit in the whole facility was obtained in the third and the fourth quarter of the year while using the correction factors recommended for all facilities (k_3mc_). Differences between the estimated mean annual values and the value obtained from measurements ranged from 80% to nearly 140% (Fig. 5). In the first quarter of the year, higher accuracy of fitting was characteristic of mean annual radon concentrations in the inhalatorium (from 95 to 99%). However, an equally good fit was obtained for the tourist section of the mine (78–82%). In both cases, better results were produced by correction factors k_3ma_ and k_3mb_ respectively (Fig. 5).

**Fig. 6:**
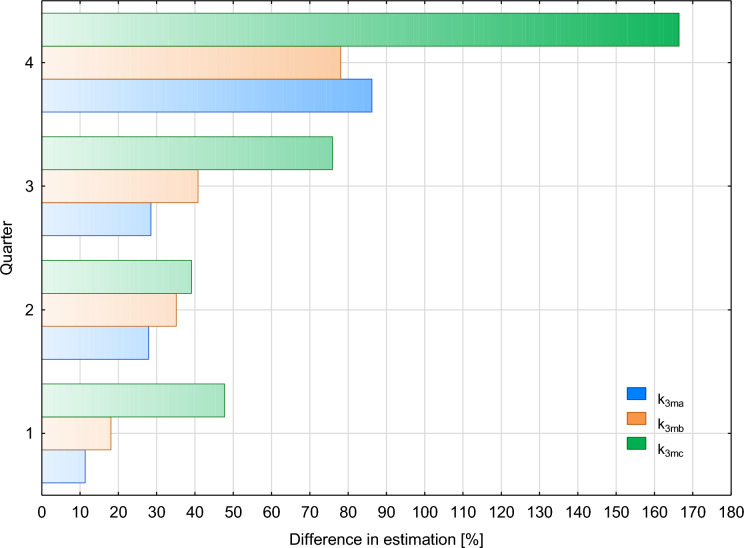
Differences in fits of mean annual measured values of radon concentration in former military facility Osówka operating as a tourist route to values estimated using quarterly correction factors k_3m_ recommended by Fijałkowska-Lichwa and Przylibski (4). Explanation: k_3ma_—quarterly correction factor for naturally ventilated facilities, k_3mb_—quarterly correction factor for facilities with a mechanical ventilation system, k_3mc_—averaged quarterly correction factor for all underground facilities. Three-month periods corresponded to four quarters of the calendar year. Exposure: 1st quarter: 2160 h, 2nd quarter: 2828 h, 3rd quarter: 2880 h, 4th quarter: 2208, colours of legend and bars are the same, for bars used a colour blur

To verify quarterly k_3m_ factors, the results of radon concentration measurements conducted at 16 measurement points in the former military facility Osówka from October 2004 to December 2005 were also used. Based on these measurements, the mean annual concentration of radon in the underground complex Osówka was determined to be 200 Bq/m^3^. To estimate the mean annual value, three groups of quarterly correction factors: k_3ma_, k_3mb_ and k_3mc_ were used. As calculations showed, the estimates best fitting the mean annual value derived from measurements are obtained in the second quarter of the calendar year, regardless of the employed k_3m_ correction factor, and in the first quarter of the year for correction factors k_3ma_ and k_3mb_. In the second quarter of the calendar year, the differences between fits obtained using particular factors are small, reaching less than 10%. In the first quarter of the year, markedly bigger differences between the estimated and the observed mean annual value were obtained using the correction factor k_3ma_. The observed differences between mean annual values converted using correction factors k_3mc_ and k_3mb_ did not exceed 10% (Fig. 6). In the third quarter of the year, fits comparable to those in the first and the second quarter of the calendar year were obtained using correction factors k_3mc_ and slightly weaker, at the level of c. 60%, using the correction factor k_3mb_. In the third quarter of the year, estimates based on the correction factor k_3ma_ were almost half as accurate (just under 30/% consistency of results). Also, in the fourth quarter of the year, the consistency of the estimated mean annual radon concentration and the value obtained from measurements was lower than in the other three quarters. Mutually comparable fits, at the level of 15–20%, between the estimated and the measured values were obtained for correction factors k_3ma_ and k_3mb_. The correction factor k_3mc_ was a rather inaccurate conversion factor for estimating mean annual concentration compared to measurement results in the fourth quarter of the calendar year (Fig. 6). The wide variation in result consistency observed in the fourth quarter of the year suggests that it is not the best time to conduct environmental measurements for estimating mean annual values.

## Conclusions

The first attempt to verify correction factors against the results of measurements conducted by an accredited dosimetry laboratory in Poland was successful. The mean annual values of radon concentration estimated using groups of monthly and quarterly correction factors recommended for underground facilities are characterized by a high goodness of fit. For a natural space (the cave) the best fit, regardless of the month in which the measurement had been performed, was obtained using correction factors k_1mb_ dedicated to non-insulated spaces (without locks, supporting structures or stoppings), and k_1mc_ dedicated to all underground facilities. In the case of the correction factor k_1ma_ intended for naturally ventilated spaces with good insulation from the atmosphere, a fit comparable to those obtained with the other two groups of one-month correction factors was obtained for measurements conducted from April to September and in November. On this basis, it was concluded that in order to estimate the mean annual radon level in the warmer part of the year (from May to September), it is better to apply the group of k_1mc_ correction factors. In such cases, the goodness-of-fit of the estimated values to those obtained from measurements is 98% in May, 82% in June, 85% in July, 88% in August, and 99% in September. When it comes to transitional periods, high fitting accuracy, at the level of 95%, was obtained using correction factors k_1mc_ in April, and k_1mb,_ k_1mc_ and k_1ma_ in November. In the cooler part of the year (from December to March), high compatibility of results was achieved using two groups of correction factors: k_1mb_ and k_1mc_. The obtained results confirm that the correction factor k_1ma_ should not be used for determining mean annual levels of radon concentration in January and February. Its application for estimating mean annual concentration based on December measurement results enables obtaining a low, 30% fit.

For a tourist facility operating in the workings of a former mine (with a large variation in concentration, reaching even an order of magnitude) a comparable fit between estimated concentrations and values obtained from measurements was obtained using three groups of correction factors, k_1ma-c_, in March and May, and for all the four groups only in December and June (about 70% fit). In September and November, the best fits were obtained using k_1ma_ correction factors, and in July and August – k_lmc_ correction factors. In the cooler part of the year (December—February) and in the transitional period (April), a better fit was obtained using two groups of correction factors: k_1mb_ and k_1mc_. Its level exceeded 90% in April and ranged between 90 and 80% in March, May June and August. The lowest accuracy (from 35% to about 2%) was observed for the estimated mean annual value based on the correction factor k_1ma_ and measurement results obtained in January, October and November.

In active mining plants equipped with mechanical ventilation systems, wide-ranging results of fitting were obtained. In the zinc and lead mining plant, a much worse fit was obtained in extraction departments than in machine depots or rooms for the staff of the ventilation department or mechanical department. In the copper mining company, the best fit of the mean annual value derived from measurement results obtained in February 2012 was obtained using k_1mc_ correction factors in the extraction departments of mine No. 1 and in the main area of mine No, 2. In the extraction departments in the western and eastern area of mine 2, the best fit was obtained using k_1md_ correction factors. The obtained results confirm that mean annual concentrations in the extraction areas of copper mining plants can be estimated using k_1mc_ and k_1md_ correction factors. In zinc and lead mining plants, on the other hand, these groups of k_1m_ correction factors provide a far better fit to measurement results obtained in departments remote from the extraction areas. Such variation in the obtained results was most likely due to the air circulation conditions. The mean level of concentration was determined on the basis of the results of environmental dosimetry measurements conducted both at workplace exposure (during effectively worked time) and during preparatory time spent in the bathroom, the lamp room, the changing room, or on the way to the workplace.

In active metal ore mines, large variations in the fit between estimated values and measurement results was observed. In copper mines, it ranged 28%-83%, and in the zinc and lead mining plant—from 58 to 98%. Therefore, the recommended choice of the optimal period of the year for conducting measurements and estimating the mean annual value in active mines ought to be verified. Such verification should be based on measurements conducted for at least a few months, and preferably one calendar year.

Along the tourist routes and in the inhalatorium, both in the former uranium mine and in the former military facility, the best fit of estimated values to those from measurements was obtained using three groups of quarterly correction factors in the second quarter of the calendar year. The biggest differences in the fit of mean annual values were observed in the third and the fourth quarter of the year for correction factors k_3mc_ recommended as averages for all underground facilities. In the first quarter of the year, a much better fit (differing by 15% to 30% from the estimated annual mean of radon concentration) was obtained using k_3ma_ and k_3mb_ factors. Hence, it was concluded that in order to determine mean annual values of radon concentration in tourist facilities located in former mines, it is best to use correction factors k_3ma_ and k_3mb_ in the first, the third and the fourth quarter of the calendar year. Additionally, when determining a mean annual concentration along a tourist route and in an inhalatorium located in a former mine, or in a military facility, one should not use correction factors k_3mc_ in the third and the fourth quarter of the calendar year. Their application is possible in the first quarter of the year, but the obtained results may differ by 45% to 55% from the real values obtained from measurements. This fit is a half to almost a fifth of that obtained when using k_3ma_ and k_3mb_ factors.

The results of the authors’ research have made it possible to indicate the optimal period (the month and quarter of the year) for conducting environmental measurements aimed at determining the mean annual value of radon concentration (Table 2). The highest accuracy of one-month estimation can be obtained while conducting environmental measurements of radon concentration in underground facilities in March, April, May and September. In three-month (quarter) periods, these are the first and the second quarter of the calendar year (Table 2). Additionally, in a natural space (a cave), it is best to estimate the mean annual value of radon concentration using two groups of correction factors: k_1mc_ and k_1mb_. When determining the mean annual value along a tourist route located in the workings of a former mine with a markedly varied level of radon concentration, it is best to use the k_1ma_ factor, recommended for naturally ventilated spaces well insulated from the atmosphere, in March and September. In April, these should be k_1mb_ and k_1mc_ correction factors. Along tourist routes and in inhalatoriums operating in the workings of former mines and in military facilities, the best period for conducting radon concentration measurements is the second quarter of the calendar year (from April to June). Due to a significantly varied range of fits between the obtained results (from 15% to more than 75% for k_3ma_ and from just above 20% to 80% for k_3mb_), the fourth quarter of the year should not be chosen for estimating the mean annual value of radon concentration. The obtained values might be less accurate compared to those obtained from measurements. This has also been confirmed by the results of research by Fijałkowska-Lichwa and Przylibski (2022).

**Table 2:** Authors’ recommendations for values of correction factors k_1m_ and k_3m_ in underground facilities relative to optimal periods for conducting environmental measurements

Type of object	Recommended exposure period	Recommended correction factors	Match compliance [%]
Cave	III	k_1mb_: 1.30 or k_1mc_:1.16	98% or 99% respectively
	IV	k_1mc_: 0.88	95%
	V	k_1mc_: 0.74	98%
	IX	k_1mc_: 0.95	99%
	XI	k_1mb_: 1.30, k_1mc_: 1.30, k_1ma_:1.20	98%, 98%, 90% respectively
	XII	k_1mb_: 1.50 or k_1mc_:1.60	95% or 97% respectively
Tourist route in mine	III	k_1ma_:1.56	90%
	IV	k_1mc_: 0.96 or k_1mb_: 0.88	98% or 90% respectively
	IX	k_1ma_: 0.63	95%
Mining company	In colder period: XII	k_1mc_ or k_1md_ 1.60	For mining area (range): 58–98%
Tourist route in abandoned uranium mine	Quarter no. 1 (January-March)	k_3ma_: 0.62	82%
	Quarter no. 2 (April-June)	k_3ma_: 0.76	82%
	Quarter no. 3 (July–September)	k_3ma_: 2.14	88%
	Quarter no. 4 (October-December)	k_3mb_: 1.31	80%
Inhalatorium in abandoned uranium mine	Quarter no. 1 (January-March)	k_3mb:_1.40, k_3ma_: 0.62	99%, 95% respectively
	Quarter no. 2 (April–June)	k_3ma:_ 0.76	90%
	Quarter no. 3 (July–September)	k_3ma:_ 2.14	95%
	Quarter no. 4 (October–December)	k_3mb_: 1.31	72%
Tourist route in military object	Quarter no. 1 (January–March)	k_3ma:_ 0.62, k_3mb:_ 1.40	89%, 82% respectively
	Quarter no. 2 (April-June)	k_3ma_: 0.76	72%
	Quarter no. 3 (July–September)	k_3ma:_ 2.14	72%
	Quarter no. 4 without recommendation	k_3m_	Too high difference of estimation

Explanation: ZG—mining plant, k_1m_ – monthly correction factor, k_3m_ – quarterly correction factor, k_3ma_ – quarterly correction factor for naturally ventilated facilities, k_3mb_ – quarterly correction factor for facilities with mechanical ventilation systems, k_3mc_ – averaged quarterly correction factor for all underground facilities, k_1ma_ – monthly correction factor for naturally ventilated facilities well insulated from the atmosphere, k_1mb_ – monthly correction factor for non-insulated facilities, k_1mc_ – averaged monthly correction factor for underground facilities, k_1md_ – monthly correction factor for facilities with mechanical ventilation systems

The results of the assessment based on archival data (years 1995–2012) provided by the Institute of Occupational Medicine in Łódź have confirmed that monthly and quarterly correction factors are a very well-suited tool for estimating mean annual radon concentrations in the warmer part of the year. This is particularly important in terms of radiation protection, as this the time when the highest risk of exposure to radon and its progeny occurs. The correction factors recommended by the authors may be successfully used to determine mean annual radon concentrations in underground facilities in other countries in Europe and worldwide located in similar climatic zones as Poland. The authors’ findings correspond to those obtained by Fijałkowska-Lichwa and Przylibski (2022).

## Data Availability

All relevant data and materials are presented in the paper.

## References

[CR1] Burke, O., & Murphy, P. (2011). Regional variation of seasonal correction factors for indoor radon levels. *Radiation Measurements.,**46*, 1168–1172. 10.1016/j.radmeas.2011.06.075 10.1016/j.radmeas.2011.06.075

[CR2] Council Directive 2013/59/Euratom of 5 December 2013 laying down basic safety standards for protection against the dangers arising from exposure to ionising radiation, and repealing Directives 89/618/Euratom, 90/641/Euratom, 96/29/Euratom, 97/43/Euratom and 2003/122/Euratom. Official Journal of The European Union. 17.1.2014, L 13/1 – L 13/73. https://eur-lex.europa.eu/eli/dir/2013/59/oj.

[CR3] Domański, T., Chruścielewski, W., & Hofman, M. (1981). Monitoring the exposure to radon decay products in mine air using passive track detectors. *Health Physics,**40*, 211–217. 6260709 10.1097/00004032-198102000-00006

[CR4] Fijałkowska-Lichwa, L., & Przylibski, T. A. (2022). Monthly and quarterly correction factors for determining mean annual radon concentration. *Environmental Geochemistry and Health*. 10.1007/s10653-022-01280-2 10.1007/s10653-022-01280-2 35501524

[CR5] Gillmore, G. K., Phillips, P. S., & Denman, A. R. (2005). The effects of geology and the impact of seasonal correction factors on indoor radon levels: A case study approach. *Journal of Environmental Radioactivity.,**84*, 469–479. 15982793 10.1016/j.jenvrad.2005.05.004

[CR6] Groves-Kirkby, C. J., Crockett, R. G. M., Denman, A. R., & Phillips, P. S. (2015). A critical analysis of climatic influences on indoor radon concentrations: Implications for seasonal correction. *Journal of Environmental Radioactivity.,**148*, 16–26. 26093853 10.1016/j.jenvrad.2015.05.027

[CR7] IAEA - International Atomic Energy Agency (2014). Radiation protection and safety of radiation sources: International Basic Safety Standards. General Safety Requirements Part 3. No. GSR Part 3. Vienna.

[CR8] ICRP - International Commission On Radiation Protection. (2011). Lung Cancer Risk from Radon and Progeny. ICRP Publication 115. Ann.

[CR9] ICRP - International Commission On Radiation Protection. (2014). Radiological protection against radon exposure. ICRP Publication 126. Ann. ICRP 43 (3). 10.1177/0146645314542212 25915928

[CR10] ICRP - International Commission On Radiation Protection. (2017). Occupational Intakes of Radionuclides: Part 3. (vol 46 No. 3/4) ICRP Publication 137.

[CR11] IRMA - International Radon Measurement Association (2017). IRMA International Industrial Guideline Industrial radon measurement guideline to get an overall view of the radon concentration in a workplace IRMA 0791–30 (website: https://www.irma-radon.org/guidelines/blog-post-title-four-pgbp8).

[CR12] Kozak, K., Mazur, J., Kozłowska, B., Karpińska, M., Przylibski, T. A., Mamont-Cieśla, K., Grządziel, D., Stawarz, O., Wysocka, M., Dorda, J., Żebrowski, A., Olszewski, J., Hovhannisyan, H., Dohojda, M., Kapała, J., Chmielewska, I., Kłos, B., Jankowski, J., Mnich, S., & Kołodziej, R. (2011). Correction factors for determination of annual average radon concentration in dwellings of Poland resulting from seasonal variability of indoor radon. *Applied Radiation and Isotopes,**69*, 1459–1465. 21652217 10.1016/j.apradiso.2011.05.018

[CR13] Krewski, D., Mallick, R., Zielinski, J., et al. (2005). Modeling seasonal variation in indoor radon concentrations. *Journal of Exposure Science & Environmental Epidemiology,**15*, 234–243. 10.1038/sj.jea.7500397 10.1038/sj.jea.7500397 15592445

[CR14] Minister of Health Announcement of 22 January 2021 concerning the National action plan in case of long-term hazards arising from exposure to indoor radon in buildings intended for human residence and workplaces. Monitor Polski, 2021, item 169.

[CR15] Müllerová, M., Mrusková, L., Holý, K., et al. (2022). Estimation of seasonal correction factor for indoor radon concentration in Slovakia: A preliminary survey. *J Radioanalytical and Nucelar Chemistry.,**331*, 999–1004. 10.1007/s10967-021-08139-3 10.1007/s10967-021-08139-3

[CR16] Olszewski J., Chruścielewski W., Jankowski J. (2005). Radon on underground tourist routes in Poland. In: International Congress Series (vol 1276, pp 360–361). Elsevier.

[CR17] Olszewski J. (2006). Exposure to radon along underground tourist routes. In: Radon w środowisku życia, pracy i nauki mieszkańców Dolnego Śląska. Polski Klub Ekologiczny, Okręg Dolnośląski. Wrocław. 55–62.

[CR18] Olszewski, J., Kacprzyk, J., & Kamiński, Z. (2010). Assessment of radiation exposure of miners to radon and its daughter products in selected non-ferrous metal mines. *Medycyna Pracy,**61*(6), 635–639. 21452566

[CR19] Olszewski, J., Zmyślony, M., Wrzesień, M., & Walczak, K. (2015). Occurrence of radon in Polish underground tourist routes. *Medycyna Pracy.,**66*(4), 557–563. 26536972 10.13075/mp.5893.00211

[CR20] Park, J. H., Lee, C. M., Lee, H. Y., & Kang, D. R. (2018). Estimation of seasonal correction factors for indoor radon concentrations in Korea. *International Journal of Environmental Research and Public Health,**15*(10), 2251. 10.3390/ijerph15102251 30326575 10.3390/ijerph15102251 PMC6210485

[CR21] Rahman, S., Mati, N., & Matiullah, G. B. M. (2007). Seasonal indoor radon concentration in the North West Frontier Province and federally administered tribal areas—Pakistan. *Radiation Measurements,**42*(10), 1715–1722. 10.1016/j.radmeas.2007.07.002 10.1016/j.radmeas.2007.07.002

[CR22] Report of Chief Sanitary Inspector GIS. (2021). Good practices for methods of measuring radon activity concentration in workplaces, buildings, premises and spaces intended for human residence. 1–33. Warszawa. Atomic Law of 29 November 2000 (Journal of Laws, 2023, Item 1173, consolidated text).

[CR50] Statistics Poland website. https://stat.gov.pl/statystyka-regionalna/jednostki-terytorialne/podzial-administracyjny-polski/?pdf=1

[CR24] Walczak, K., Olszewski, J., Politański, P., & Zmyślony, M. (2017). Occupational exposure to radon for underground tourist routes in Poland doses to lung and the risk of developing lung cancer. *International Journal of Occupational Medicine and Environmental Health.,**30*(5), 687–694. 10.13075/ijomeh.1896.00987 28584312 10.13075/ijomeh.1896.00987

